# Association of high lipoprotein (a) level with carotid atherosclerosis and all-cause mortality

**DOI:** 10.1016/j.ajpc.2025.101023

**Published:** 2025-06-01

**Authors:** Anthony Matta, Dorota Taraszkiewicz, Pauline Cougoul, Sylvie Lemozy, Jean Ferrières

**Affiliations:** a Department of cardiology, Toulouse Rangueil University Hospital, and UMR INSERM 1295, TSA 50032 Toulouse, France; b Department of cardiology, Centre Hospitalier Intercommunal des Vallées de l'Ariège, Saint Jean de Verges, France; c Notre Dame des Secours University Hospital Center, Street 93, Byblos, Postal Code 3, Lebanon / School of Medicine and Medical Sciences, Holy Spirit University of Kaslik, P.O.Box 446, Jounieh, Lebanon; d Department of Endocrinology and Diabetology, Toulouse Rangueil University Hospital, TSA 50032 Toulouse, France

**Keywords:** Lipoprotein (a), All-cause mortality rate, Atherosclerosis

## Abstract

**Background:**

Lipoprotein (a) Lp(a) is a genetically inherited low-density lipoprotein like particle with proinflammatory, prothrombotic and proatherogenic properties. Current guidelines recommend at least one-time measurement of Lp(a) in cardiovascular risk assessment in each adult person’s lifetime.

**Aims:**

The present study evaluates the association between exposure to a high circulating level of Lp(a) (≥50mg/dl or ≥125nmol/L) and atherosclerosis and all-cause of death after a very long-term follow-up.

**Methods:**

A retrospective analysis was performed on 11,990 subjects admitted to the department of preventive cardiology at Toulouse University Hospital, Rangueil, France and who underwent at least one Lp (a) measurement between September 1995 and December 2023. The living status (alive or dead) of each of study’s participants was assessed by March 2024. The study population was divided into two groups: first, according to Lp (a) level (normal versus high circulating Lp (a) level), and second, according to the living status.

**Results:**

High-Lp(a) level group includes 3195 participants. They were significantly more women (47.5 % vs.41.4 %), non-obese (89.1 % vs.84.7 %), sportier (71 % vs. 68.1 %) and normotensive (70.5 % vs. 67.6 %). The adjusted logistic regression on traditional cardiovascular risk factors reveal a positive association between High Lp(a) level and atherogenic findings on doppler ultrasound of carotid arteries [ORa = 1.308;95 %CI(1.182–1.447), *p* = 0.001]. The all-cause mortality rate was comparable between the two study groups over 13 years of follow-up (7.4 % vs. 8 %). Survival analyses with Kaplan-Meier curves (*p* = 0.643) and Cox regression [HR = 0.981, 95 %CI (0.844–1.141), *p* = 0.811] showed no significant difference in survival outcomes.

**Conclusion:**

Long-term exposure to high Lp(a) is a predictor for atherosclerotic cardiovascular disease, but not significantly associated with risk of death from any cause.

## Lay summary

The dosage of Lp(a) seems informative in healthy adults with atherosclerotic findings.
  - A high Lp(a) level is a marker of carotid atherosclerosis even though in the absence of traditional cardiovascular risk factors.
  - No association exists between Lp(a) and all-cause of death after very-long term of follow up.

## Graphical abstract

Illustration of the association between a high Lp(a) level with carotid atherosclerosis, but not with all-cause mortality.

**Figure:**
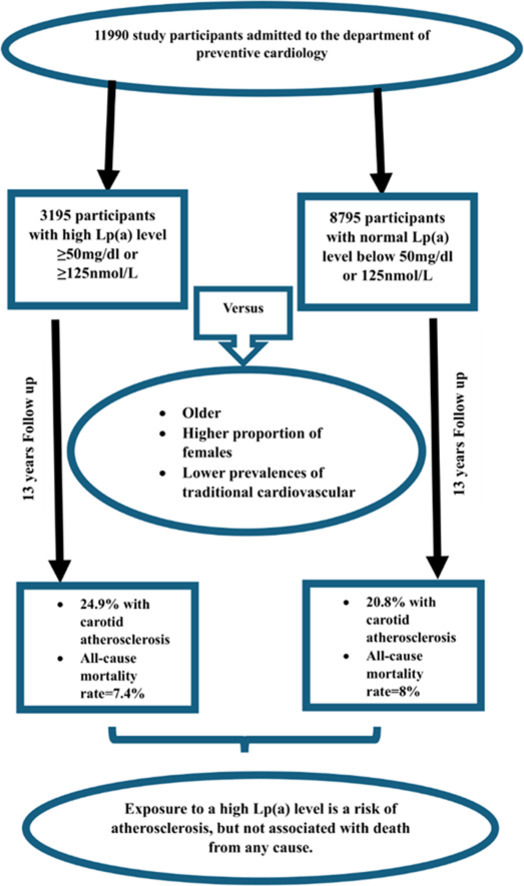

## 1 Introduction

Lipoprotein a [Lp(a)] is a genetically inherited low-density lipoprotein like particle with proinflammatory, prothrombotic and proatherogenic properties. It consists of an apolipoprotein B-100 linked by a disulfide bridge to apolipoprotein (a) [1]. Existing literature provides strong evidence to establish a high Lp(a) concentration as a risk factor for atherosclerotic cardiovascular disease. Indeed, Lp(a) is involved in the development of coronary artery disease, aortic valve stenosis and cerebrovascular disease [[2], [3], [4], [5], [6], [7]]. The 2022 Lp(a) consensus statement of the European Atherosclerosis Society has settled a threshold value at 50mg/dl or 125nmol/L to “rul-in” risk [8]. This risk-enhancing cut-off has been endorsed by the American, European and Canadian cardiovascular societies as well as by the National Lipid Association [9,10]. Current guidelines recommend at least one-time measurement of Lp(a) in cardiovascular risk assessment in each adult person’s lifetime [9]. The only approved therapy by the FDA (food and drug administration) for high circulating Lp(a) is lipoprotein apheresis. It is approved for patients with familial hypercholesterolemia, an LDL-c level ≥100mg/dl and a Lp(a) ≥60mg/dl. The last years have witnessed a growing attention on the clinical value of Lp(a) which has become more prominent in most guidelines. New therapy with short interfering RNA agent targeting the LPA gene transcription is being developed [9]. While a robust link exists between high circulating Lp(a) levels and atherosclerotic plaque development, the evidence concerning its association with the risk of cardiovascular mortality either in general healthy population or in patients with chronic diseases is not as conclusive. A considerable number of studies revealed an increased cardiovascular mortality rate in the setting of elevated Lp(a) [11] while no significant association has been showed in others [12,13]. Till now, it remains uncertain if lowering Lp(a) level reduces cardiovascular events [8]. Studies assessing the association of circulating Lp(a) and the risk of death from any cause are still scarce. Thus, the precise impact of Lp(a) per itself on all-cause mortality remains uncertain. To address this question, the present hospital based retrospective cohort study assesses the effect of a high circulating Lp (a) level on all-cause of death and atherosclerotic findings on carotid arteries in a general population after a very long-term follow-up.

## 2 Materials and methods

### 2.1 Study design and population

This cohort is a retrospective analysis of collected data within a dedicated database that includes patients admitted to a day hospital of the department of preventive cardiology at Toulouse University Hospital, Rangueil, France for a primary prevention and cardiovascular risk classification. All patients who underwent at least one Lp (a) measurement between September 1995 and December 2023 were screened. Patients with incomplete data for Lp (a) measurement (*N* = 370) or for living status (*N* = 200) were excluded from this study. According to guidelines, a plasma circulating level of Lp (a) ≥ 50mg/dl or 125nmol/l was considered high. The living status (alive or dead) of each of study’s participants was assessed by March 2024. The study population was divided into two groups: first, according to Lp (a) level (normal versus high circulating Lp (a) level), and second, according to the living status. Then, we identified the profile of patients with high Lp (a) level and determined independent predictors of long-term mortality evaluating the association between plasma Lp (a) concentration and survival. Patients were informed at hospital admissions that their clinical data could be used for research purposes in anonymized form, and non-opposition consent forms were signed and obtained. The cohort was registered by the Ministry of Research and the Regional Health Agency Occitanie (no. DC-2017–298). Our institution does not require ethics approval for observational retrospective cohort.

### 2.2 Data collection and endpoint

Data concerning baseline characteristics of study participants, cardiovascular risk factors, full lipid panel tests [total cholesterol, LDL-c, HDL-c, triglycerides, Apo B, Apo A1, Lp (a)] and carotids doppler ultrasound results were retrospectively collected. The analyses of venous blood samples and carotids doppler ultrasound were performed during the index hospital stay at the department of preventive cardiology. The Roche-Cobas 8000 analyzer with immunoassay diagnostic tests was used to precisely quantify the circulating Lp (a) level since 2013. This analyzer uses “electrochemiluminescence sandwich assays based on biotin and streptavidin interactions. The analyte and antibody form a sandwich, and magnetic particles coated with streptavidin are then added. The free biotin prevents some of the antibody-analyte sandwich from binding to the microparticle by binding to streptavidin-coated microparticles instead” [14]. At early stage, total Lp(a) level was determined by an enzyme-linked immunosorbent assay using sheep polyclonal monospecific antibodies against purified human Lp(a) [15]. The living status of study participants were observed by March 2024 in a French register of deaths. We searched the living status using the full name and the date of birth of each study participant in the database of the death register, then finding out who has died and the date of death. The date of Lp (a) measurement is considered the date of inclusion. Obesity was defined by a body mass index ≥30kg/m². Hypertension was defined according to ESC guidelines as a confirmed office systolic blood pressure ≥140 mmHg or diastolic blood pressure ≥90 mmHg) [16]. Sport was defined as exercising a minimum of 2 h per week of physical activity. Smoking status was defined as current smoking (Yes/No). The glomerular filtration rate was calculated based on Cockroft-Gault formula (1.23 x weight(kg) x (140-age)/creatinine(μmol/l) for men, 1.04 x weight(kg) x (140-age)/creatinine (μmol/l) for women). Continuous-wave doppler ultrasound images and duplex ultrasound were used to assess the lumen area, intima-media thickness, adventitia and blood flow velocity in the common, internal and external carotid arteries. These measurements can estimate the diameter of the vessels, thereby determining the presence of atherosclerotic plaque and the amount of obstruction. In this study, carotid artery atherosclerosis was defined by the detection of any plaque on carotid doppler ultrasound. The primary endpoint of this study was to assess if people with a high plasma Lp (a) level are at increased risk of death from any cause. The secondary end point was to evaluate the association between a high plasma Lp(a) level and carotid atherosclerosis.

### 2.3 Statistical analysis

Categorical variables are expressed by numbers and percentages and compared using the Chi square test. The continuous variables are summarized by means and standard deviations and compared using the Student’s *t*‐test. The normality and homoscedasticity tests for quantitative variables were performed. Multivariate logistic regression models were performed to identify the characteristics of patients with high Lp (a) level and the association between the presence of carotid plaque and a high Lp(a) level. Cumulative survival curves for total survival were drawn using the Kaplan–Meier method, and the log‐rank test was used for survival analysis between groups (high versus normal Lp (a) level). A Cox proportional hazard model with multivariable analyses including variables that were thought to be independently associated with mortality was performed. These variables include age, sex, obesity, systemic hypertension, diabetes mellitus, smoking, exercise, Lp(a) level as dichotomous variable (high versus low) and as continuous variable, carotid atherosclerosis, glomerular filtration rate, and apolipoproteins A1 and B, HDL-c and LDL-c levels. A two‐sided *P*‐value <0.05 was considered statistically significant. Statistical analyses were carried out using SPSS version 20 (IBM Corp., Armonk NY, USA).

## 3 Results

A total of 11,990 patients were included in this cohort. The mean age of study participants is 57.2 ± 12 years and 43.1 % are women. Table 1 showed the characteristics of study population stratified by Lp (a) level. Patients with elevated Lp (a) level were older (57.6 vs 57.1 years, *p* = 0.001) and have significantly lower rates of different cardiovascular risk factors but higher proportion of females (47.5 % vs 41.4 %, *p* = 0.001). The means of total cholesterol (236±57 vs. 227±53 mg/dl), HDL-c (59±24 vs. 56±19mg/dl), LDL-c (152±52 vs145±49mg/dl), Apo A1 (154±35 vs. 151±31mg/dl) and Apo B (121±42 vs. 116±35mg/dl) were also higher in this group of patients. Atherosclerosis of carotid artery was more detected in patients with high Lp(a) level (24.9 % vs. 20.8 %, *p* = 0.001). The adjusted multivariate logistic regression revealed significant positive associations between high Lp(a) level and atherosclerosis [ORa = 1.308;95 %CI(1.182–1.447),*p* = 0.001], female gender [ORa = 1.224;95 %CI(1.122–1.335), *p* = 0.001] and sport [ORa = 1.116;95 %CI(1.018–1.225),*p* = 0.019), but negative associations with hypertension [ORa = 0.907, 95 %CI(0.825–0.997), *p* = 0.045] and obesity [ORa = 0.727, 95 %CI(0.638–0.829), *p* = 0.001] (Table 2). The association between the presence of carotid plaque and a high Lp(a) level independently from traditional cardiovascular risk factors was confirmed by another multivariable logistic regression model [ORa = 1.314; 95 %CI(1.188–1.459), *p* = 0.001] (Table 3).

**Table 1:** Characteristics of study population stratified by lipoprotein (a) level.

	**Study population (*N*=11,990)**	**Low Lp (a) <50mg/dl (*N*=8795)**	**High Lp (a) ≥50mg/dl (*N*=3195)**	**p-value**
**Age (Year)**	57.2 ± 12	57.1 ± 12	57.6 ± 12	0.0287
**Females (n, %)**	5163(43.1 %)	3644(41.4 %)	1519 (47.5 %)	0.001
**Obese (n, %)**	1689(14.1 %)	1344(15.3 %)	345(10.8 %)	0.001
**Systemic hypertension (n, %)**	3793(31.6 %)	2850(32.4 %)	943(29.5 %)	0.003
**Diabetes mellitus (n, %)**	781(6.5 %)	600(6.8 %)	181(5.7 %)	0.023
**Smoking (n, %)**	1905(15.9 %)	1415(16.1 %)	490(15.3 %)	0.319
**Sport (≥2 h/week)**	8258(68.9 %)	5990(68.1 %)	2268(71 %)	0.003
**GFR (ml/min/1.73m²)**	95±51	96±55	92±37	0.001
**Cholesterol total (mg/dl)**	229±54	227±53	236±57	0.001
**HDL-c (mg/dl)**	57±21	56±19	59±24	0.001
**LDL-c (mg/dl)**	147±50	145±49	152±52	0.001
**Triglycerides (mg/dl)**	131±75	134±77	124±67	0.001
**Apo A1 (mg/dl)**	152±32	151±31	154±35	0.001
**Apo B (mg/dl)**	117±37	116±35	121±42	0.001
**Carotid atherosclerosis (n, %)**	2627(21.9 %)	1833(20.8 %)	794(24.9 %)	0.001
**Rest heart rate (bpm)**	67±12	67±12	66±12	0.194
**Follow up (year)**	13±8	13±8	13±7.8	0.575
**Death (n, %)**	935(7.8 %)	700(8 %)	235(7.4 %)	0.276

*GFR = glomerular filtration rate; HDL-*c* = high density lipoprotein cholesterol; LDL-*c* = low density lipoprotein cholesterol; Apo A1 = apolipoprotein A; Apo *B* = apolipoprotein B.

**Table 2:** Multivariable logistic regression identifying factors associated with high lipoprotein (a) level.

	**OR**	**95 %CI**	**p-value**
**Age**	1.002	[0.998–1.006]	0.228
**Women**	1.224	[1.122–1.335]	0.001
**Obese (BMI≥30kg/m²)**	0.727	[0.638–0.829]	0.001
**Systemic hypertension (SBP≥140mmHg or DBP≥90mmHg)**	0.907	[0.825–0.997]	0.045
**Diabetes mellitus**	0.956	[0.799–1.144]	0.625
**Sport (›2hours/week)**	1.116	[1.018–1.225]	0.019
**Mean total cholesterol**	1.020	[0.801–1.1298]	0.872
**Mean LDL-c**	1.294	[0.999–1.677]	0.051
**Carotid atherosclerosis**	1.308	[1.182–1.447]	0.001
**All-cause mortality**	0.941	[0.804–1.101]	0.449

*BMI = body mass index; SBP = systolic blood pressure; DBP = diastolic blood pressure).

**Table 3:** Multivariable logistic regression assessing the association between the presence of carotid plaque and high Lp(a) level after adjusting on traditional cardiovascular risk factors.

	**OR**	**95 %CI**	**p-value**
**High Lp(a) (≥50mg/dl)**	1.314	[1.188–1.459]	0.001
**Age**	1.062	[1.057–1.067]	0.001
**Men**	1.741	[1.580–1.919]	0.001
**Obese (BMI≥30kg/m²)**	0.834	[0.726–0.959]	0.011
**Systemic hypertension (SBP≥140mmHg or DBP≥90mmHg)**	1.392	[1.262–1.536]	0.001
**Diabetes mellitus**	1.595	[1.349–1.887]	0.001
**Sport (›2hours/week)**	0.882	[0.804–0.967]	0.008
**Mean LDL-c**	1.042	[0.944–1.151]	0.407
**Smoking**	1.289	[1.170–1.419]	0.001

*BMI = body mass index; SBP = systolic blood pressure; DBP = diastolic blood pressure).

**Fig. 1:**
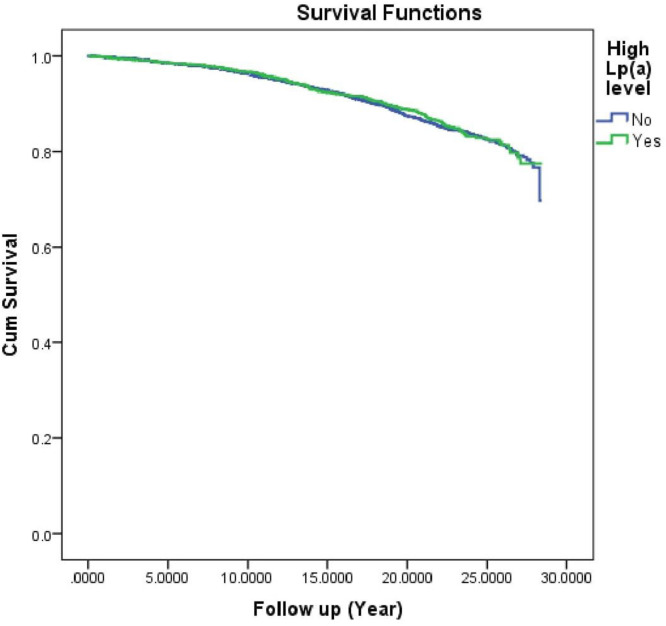
Kaplan Meier curves showed no significant differences in survival over time between high versus normal Lp(a) level (log rank test, *p* = 0.643).

Over almost 13 years of follow up, 7.8 % (*N* = 935) of study participants died (Table 5). All-cause mortality rate was comparable between the two study groups of patients with high versus normal Lp (a) level (7.4 % vs 8 %, *p* = 0.276) (Table 1). This result was confirmed by Kaplan Meier curves that also showed no difference in survival outcome according to Lp (a) level (logrank test, *p* = 0.645) (Fig. 1). Moreover, Cox regression model failed to identify high Lp(a) level as a predictor of death from any cause [HR = 0.979; 95 %CI(0.842–1.138), *p* = 0.787]. The result was similar when Lp(a) was analysed as continuous variable [HR = 1.013;95 %CI(0.973–1.053), *p* = 0.519]. However, it revealed carotid atherosclerosis as a risk factor for all-cause mortality [HR = 1.384; 95 %CI(1.195–1.602), *p* = 0.001] (Table 5). As expected, survivors were younger (57±12 vs. 59±11 years), more females (44.1 % vs. 31 %), sportier (70.5 % vs. 50.2 %) and have less cardiovascular risk factors including obesity (13.8 % vs. 17.5 %), systemic hypertension (31.1 % vs. 38.5 %), diabetes mellitus (6.2 % vs. 10 %), and smoking (15.7 % vs. 18.2 %) (Tables 4).

**Table 4:** Characteristics of study population stratified by living status.

	**Study population (*N*=11,990)**	**Dead participants (*N*=935)**	**Survivors (*N*=11,055)**	**p-value**
**Age (Year)**	57.2 ± 12	59±11	57±12	0.001
**Females (n, %)**	5163(43.1 %)	290(31 %)	4873(44.1 %)	0.001
**Obese (n, %)**	1689(14.1 %)	164(17.5 %)	1525(13.8 %)	0.002
**Systemic hypertension (n, %)**	3793(31.6 %)	360(38.5 %)	3433(31.1 %)	0.001
**Diabetes mellitus (n, %)**	781(6.5 %)	93(10 %)	688(6.2 %)	0.001
**Smoking (n, %)**	1905(15.9 %)	170(18.2 %)	1735(15.7 %)	0.046
**Sport (≥2 h/week)**	8258(68.9 %)	469(50.2 %)	7789(70.5 %)	0.001
**GFR (ml/min/1.73m²)**	95±51	90±38	95±52	0.003
**Cholesterol total (mg/dl)**	229±54	229±49	229±54	0.924
**HDL-c (mg/dl)**	57±21	54±16	57±21	0.001
**LDL-c (mg/dl)**	147±50	147±45	146±50	0.741
**Triglycerides (mg/dl)**	131±75	141±77	130±74	0.001
**Apo A1 (mg/dl)**	152±32	147±29	152±32	0.001
**Apo B (mg/dl)**	117±37	118±30	117±37	0.387
**Lp (a) (mg/dl)**	42±19	39±12	43±19	0.609
**High Lp (a) (n, %)**	3195(26.6 %)	235(25.1 %)	2960(26.8 %)	0.276
**Carotid plaque (n, %)**	2627(21.9 %)	303(32.4 %)	2324(21 %)	0.001
**Rest heart rate (bpm)**	67±12	66±12	67±12	0.159
**Follow up (year)**	13±8	12.5 ± 7	13±8	0.061

*GFR = glomerular filtration rate; HDL-*c* = high density lipoprotein cholesterol; LDL-*c* = low density lipoprotein cholesterol; Apo A1 = apolipoprotein A; Apo *B* = apolipoprotein B; Lp(a) = lipoprotein (a).

**Table 5:** The multivariable Cox proportional-hazards regression survival model with lipoprotein (a) as dichotomous variable (high versus low) and as continuous variable.

	HR*	**95 %CI**	**p-value**
**Age**	1.045	[1.038–1.053]	0.001
**Women**	0.620	[0.530–0.726]	0.001
**Obesity**	1.117	[0.929–1.342]	0.237
GFR*	1.043	[0.915–1.189]	0.523
**Systemic hypertension**	1.065	[0.926–1.226]	0.373
**Diabetes Mellitus**	1.161	[0.923–1.460]	0.200
**Smoking**	1.054	[1.926–1.201]	0.421
**Sport (≥2Hours/week)**	0.986	[0.872–1.115]	0.829
**High Lp(a) level (≥50mg/dl)**	0.979	[0.842–1.138]	0.787
**Atherosclerosis on carotid artery**	1.384	[1.195–1.602]	0.001
**HDL-c level (mg/dl)**	0.626	[0.385–1.017]	0.059
**LDL-c level(mg/dl)**	0.964	[0.829–1.120]	0.635
	HR*	**95 %CI**	**p-value**
**Age**	1.045	[1.038–1.053]	0.001
**Women**	0.619	[0.529–0.725]	0.001
**Obesity**	1.119	[0.931–1.345]	0.228
GFR*	1.043	[0.915–1.190]	0.520
**Systemic hypertension**	1.066	[0.926–1.226]	0.369
**Diabetes Mellitus**	1.161	[0.924–1.460]	0.199
**Smoking**	1.055	[0.926–1.202]	0.418
**Sport (≥2Hours/week)**	0.985	[0.871–1.113]	0.811
**Lp(a) level (mg/dl)**	1.013	[0.973–1.053]	0.519
**Atherosclerosis on carotid artery**	1.383	[1.194–1.601]	0.001
**HDL-c level (mg/dl)**	0.623	[0.384–1.013]	0.056
**LDL-c level(mg/dl)**	0.961	[0.828–1.117]	0.610

⁎ Hazard ratio (HR); GFR = glomerular filtration rate; Lp(a) = lipoprotein a; HDL-*c* = high density lipoprotein cholesterol; LDL-*c* = low density lipoprotein cholesterol.

## 4 Discussion

The main findings of this large cohort with very long-term follow-up are summarized as follows: a high Lp(a) level is a risk factor for carotid atherosclerosis but not associated with survival/all-cause of death; women and sporty non-obese normotensive people are more likely to have a high Lp(a) level.

Whilst traditional cardiovascular risk factors were more common in patients with a normal Lp(a) level, the all-cause mortality rate was almost similar than that in patients with a high Lp(a) level. At the first instance, one can speculate that Lp(a) may indirectly increase the risk of death from any cause but not strongly enough to counterbalance the effect of cardiovascular risk factors. However, neither the adjusted logistic regression nor the Cox hazard regression analysis and Kaplan Meier curves have revealed a significant difference in survival outcome related to a high Lp(a) level. In line with our result, a published study by Zewinger et al. didn’t found a significant association between Lp (a) concentration and all-cause and cardiovascular mortality in a group of patients with established coronary artery disease [17]. A similar result was also revealed by two prospective cohorts on type 2 diabetic patients [18]. Moreover, a large study including 72,683 participants have failed to show a significant association between Lp(a) level and non-vascular mortality [19]. Results from BiomarCARE consortium on European population, a population-based cohort, showed no significant association between Lp(a) concentration and all-cause mortality [20]. This similar finding strongly supports the conclusion of our non-population-based cohort. An analysis of ECAD registry reported a synergic interaction between Lp(a) and LDL-c by observing an increased long-term mortality in patients with coronary artery disease and high Lp(a) level, but only when LDL-c is above 100mg/dl [21]. Lastly, two meta-analyses have concluded that high Lp(a) level increased the risk of cardiovascular events, but without a significant association with cardiovascular and all-cause mortality [[22], [23]]. On the contrary, a recent Mendelian randomization study suggested a positive association between Lp(a) and cardiovascular and digestive mortality [24]. Few studies have summarized the association between Lp(a) and risk of death from any cause [11,[25], [26]]. The prothrombotic, anti-fibrinolytic and proatherogenic roles of Lp(a) might interfere in the pathophysiology of myocardial infarction and atherosclerosis leading to increase the cardiovascular mortality which may rise all-cause mortality in return. So, till now, the mechanisms by which Lp(a) may affect non-vascular mortality remain unrecognized. This fact also explains the inconsistency of existing literature derived from the non-uniform conclusions of different studies. Finally, we may conclude that in countries were cardiovascular mortality is low like France, the impact of Lp(a) on all-cause mortality tends to be weak as shown by the present study. The conditional association between total mortality and Lp(a) (only in coronary artery disease patients with uncontrolled LDL-c) revealed by ECAD registry enrolling patients undergoing coronary angiography may represent another example.

Conversely, existing evidence linking high Lp(a) level with atherosclerosis are more consistent. Results of our study support the guidelines defining an absolute risk threshold of 50mg/dl or 125nmol/l for Lp(a) proatherogenic property [[9], [10],^27^]. The present study showed a positive association between Lp(a) level ≥50mg/dl and atherosclerotic findings on carotid artery independently from traditional cardiovascular risk factors. The Lp (a) induces vascular inflammation, thrombosis, atherogenesis and calcification [27]. The proatherogenic property of Lp(a) ensues from the similarity of apo B moiety of Lp(a) to LDL particles [28]. Although, Lp(a) carries the oxidized phospholipids that upregulate monocyte cytokine expression and fibrin/fibrinogen and endothelial cells interaction [28]. The physiological role of Lp(a) is not yet fully understood, but the three defined roles of Lp(a) in progression of the atherosclerotic process include its proatherogenic, prothrombotic, and pro-inflammatory effects. The proatherogenic effect is related to Apolipoprotein B-100 that increases foam cell formation, smooth muscle cell proliferation and adhesion molecules production. The prothrombotic effect is linked to apolipoprotein (a) that inhibits plasminogen activation and enhances platelet activation and tissue factor pathway inhibitor activity. The pro-inflammatory effect results from the production of IL-6 and TNF-α [29].

Lastly, we believe that the present study helps to fill the knowledge gap on the link between Lp (a) and all-cause mortality, supports proatherogenic property of Lp(a) and identifies the subset of population at risk for a high Lp (a) level exposure.

### 4.1 Limitations

The present cohort is a non-population-based study, and its design may be predisposed to selection bias. However, the homogeneity of our conclusion with that of a population-based cohort (BiomarCARE) strengthens the study findings. We reported all-cause mortality because we are not able to identify the cause of death in all study participants, and subsequently, we are also unable to report cause-specific mortality. Subsequent studies should be prospective and should include cardiovascular death, stroke, coronary heart disease and other peripheral atherosclerosis as endpoints in addition to all-cause mortality. The dosage of apolipoprotein (a) isoforms was not performed making difficult to unmask a possible association between smaller apolipoprotein (a) isoforms and all-cause mortality. The risk of all-cause mortality should differ largely between patients recruited earlier and those recruited more recently. Also, the medical treatment represents a key confounder for the survival outcomes as it differs among participants and varies over time. Study participants may develop cardiovascular events during the follow up period which may subsequently have a remarkable impact on prognosis and affect the risk of all-cause mortality. We are not able to quantitate plaque and provide analyses regarding whether Lp(a) related to the extent/severity of plaque. We highlight that analyse results using continuum of Lp (a) level rather than dichotomized Lp(a) level and with different thresholds of Lp(a) level were comparable.

## 5 Conclusion

A high circulating Lp(a) concentration is a predictor of atherosclerotic cardiovascular disease but not associated with the risk of death from any cause. The subset of population who is likely exposed to high Lp(a) level includes women and sporty, non-obese and normotensive persons. Clinical trials to assess the additive value of Lp(a) target therapy in the management of adverse cardiovascular events in the setting of primary and/or secondary prevention may seem interesting for this high-risk population.

## Author agreement statement

We the undersigned declare that this manuscript is original, has not been published before and is not currently being considered for publication elsewhere. We confirm that the manuscript has been read and approved by all named authors and that there are no other persons who satisfied the criteria for authorship but are not listed. We further confirm that the order of authors listed in the manuscript has been approved by all of us. We understand that the Corresponding Author is the sole contact for the Editorial process. He is responsible for communicating with the other authors about progress, submissions of revisions and final approval of proofs Signed by all authors as follows:Anthony Matta

-Dorota Taraszkiewicz
-Pauline Cougoul
-Sylvie Lemozy
-Jena Ferrières

## Funding source

None.

## CRediT authorship contribution statement

**Anthony Matta:** Writing – review & editing, Writing – original draft, Visualization, Validation, Methodology, Formal analysis, Data curation, Conceptualization. **Dorota Taraszkiewicz:** Visualization, Validation, Data curation, Conceptualization. **Pauline Cougoul:** Visualization, Validation, Data curation, Conceptualization. **Sylvie Lemozy:** Visualization, Validation, Data curation, Conceptualization. **Jean Ferrières:** Writing – original draft, Visualization, Validation, Supervision, Methodology, Formal analysis, Data curation, Conceptualization.

## Declaration of competing interest

None.
